# A Holistic Approach to Geriatric Rehabilitation: Leveraging the MFOA for Superior Patient Care

**DOI:** 10.1093/geroni/igaf122.2444

**Published:** 2025-12-31

**Authors:** Gregory Hartley, Susan Leach, Kenneth Miller, Myla Quiben, Laura Gras, Marni Larkin, Michelle Lusardi

**Affiliations:** University of Miami, Coral Gables, Florida, United States; University of New Mexico, Albuquerque, New Mexico, United States; Medical University of South Carolina, Charleston, South Carolina, United States; The University of North Texas Health Science Center at Fort Worth, Fort Worth, Texas, United States; Ithaca College, Ithaca, New York, United States; Gyrotonic Manhasset, Manhasset, New York, United States; Sacred Heart University, Fairfield, Connecticut, United States

## Abstract

The Movement Framework for Older Adults (MFOA) integrates the Geriatric 5Ms (Mind, Mobility, Medications, Multicomplexity, Matters Most) and the International Classification of Functioning, Disability, and Health (ICF) to provide a comprehensive approach to geriatric rehabilitation. This study provides a comparative analysis of two patients with Parkinson’s disease, demonstrating MFOA’s application in addressing their unique needs within a physical therapy context. The Geriatric 5Ms serve as the lens through which patient assessments and interventions are tailored, focusing on what is most important to the individual. The ICF model further supports this by categorizing health and health-related domains, ensuring a holistic perspective that considers all aspects of a patient’s well-being. MFOA emphasizes individualized care plans, incorporating interventions and tasks specific to each patient’s goals. This approach ensures that both physical and mental health are addressed to achieve optimal outcomes. The framework’s application involves a thorough assessment of the patient’s physical capabilities, psychological state, and social context, allowing for a comprehensive and patient-centered care plan. The integration of the Geriatric 5Ms and ICF models within the MFOA framework ensures that the care provided is aligned with what matters most to the patient, enhancing quality of life and engagement in therapy. This work underscores the novelty and utility of the MFOA in physical therapy management of older adults. By combining the Geriatric 5Ms and the ICF, the MFOA offers a unique and comprehensive approach that addresses the multifaceted needs of older adults, potentially improving geriatric rehabilitation outcomes through personalized, holistic care.

